# Rural Community Knowledge of Stroke Warning Signs and Risk Factors

**Published:** 2005-03-15

**Authors:** Todd S Harwell, Lynda L Blades, Carrie S Oser, Crystelle C Fogle, Steven D Helgerson, Dorothy Gohdes, Dennis W Dietrich, Anne M Burnett, Nicholas J Okon, Martha J Allen, Daniel V Rodriguez, Joseph A Russell

**Affiliations:** Montana Department of Public Health and Human Services; Montana Department of Public Health and Human Services, Helena, Mont; Montana Department of Public Health and Human Services, Helena, Mont; Montana Department of Public Health and Human Services, Helena, Mont; Montana Department of Public Health and Human Services, Helena, Mont; Montana Department of Public Health and Human Services, Helena, Mont; Benefis Healthcare, Great Falls, Mont; Benefis Healthcare, Great Falls, Mont; St Vincent Healthcare, Billings, Mont; St Vincent Healthcare, Billings, Mont; Deaconess Billings Clinic, Billings, Mont; City of Great Falls Fire and Rescue, Great Falls, Mont

## Abstract

**Introduction:**

Rapid identification and treatment of ischemic stroke can lead to improved patient outcomes. Public education campaigns in selected communities have helped to increase knowledge about stroke, but most data represent large metropolitan centers working with academic institutions. Much less is known about knowledge of stroke among residents in rural communities.

**Methods:**

In 2004, 800 adults aged 45 years and older from two Montana counties participated in a telephone survey using unaided questions to assess awareness of stroke warning signs and risk factors. The survey also asked respondents if they had a history of atrial fibrillation, diabetes, high blood pressure, high cholesterol, smoking, heart disease, or stroke.

**Results:**

More than 70% of survey participants were able to correctly report two or more warning signs for stroke: numbness on any side of the face/body (45%) and speech difficulties (38%) were reported most frequently. More than 45% were able to correctly report two or more stroke risk factors: smoking (50%) and high blood pressure (44%) were reported most frequently. Respondents aged 45 to 64 years (odds ratio [OR] 2.44; 95% confidence interval [CI], 1.78–3.46), women (OR 2.02; 95% CI, 1.46–2.80), those with 12 or more years of education (OR 1.96; 95% CI, 1.08–3.56), and those with high cholesterol (OR 1.68; 95% CI, 1.17–2.42) were more likely to correctly identify two or more warning signs compared with respondents without these characteristics. Women (OR 1.48; 95% CI, 1.07–2.05) and respondents aged 45 to 64 years (OR 1.35; 95% CI, 1.01–1.81) were also more likely to correctly identify two or more stroke risk factors compared with men and older respondents.

**Conclusion:**

Residents of two rural counties were generally aware of stroke warning signs, but their knowledge of stroke risk factors was limited.

## Introduction

Public health efforts to promote stroke awareness and the need to seek urgent treatment have assumed a new importance in the years since the publication of a major clinical trial showing decreased short-term disability and improved outcomes for patients experiencing an ischemic stroke after thrombolytic therapy (1). Prehospital barriers to prompt treatment for ischemic stroke include the lack of awareness of stroke warning signs in patients and family members, underuse of 911 emergency medical services (EMS), and long distances to tertiary-care facilities that provide diagnostic and treatment services (2-5). These barriers can lead to delayed presentation to the emergency department and to ineligibility for time-dependent treatment. Achieving increased use of thrombolytic therapy within the three-hour window in one community required a multilevel intervention to influence the knowledge and behavior of the public, the response of EMS, and the coordination of diagnostic and treatment facilities at the hospitals (6,7).

From a public health perspective, an important component for the success of stroke interventions is to improve public knowledge about stroke, particularly focusing on individuals at high risk and their family members and caregivers. Public education campaigns in selected communities have been effective in increasing the level of knowledge about stroke, but most data have come from large metropolitan centers working with academic institutions (7,8). Much less is known about basic knowledge of stroke symptoms, risk factors, and the need for urgent intervention among residents in rural communities that are relatively isolated from major metropolitan centers.

We conducted a telephone survey in two rural counties in Montana in 2004. This report describes the level of awareness of stroke warning signs and risk factors and the public perception of the need to call 911 EMS for stroke in residents aged 45 years and older.

## Methods

### Setting

The population for this study included residents living in Cascade and Yellowstone counties, which include the cities of Great Falls (Cascade County) and Billings (Yellowstone County). The 2000 census population for Cascade County was 80,357 (9). Fourteen percent of the population was aged 65 years and older and 23% was aged 45 to 64 years. The majority of residents were white (91%) or American Indian (4%). The 2000 census population for Yellowstone County was 129,352. Thirteen percent of the Yellowstone County population was aged 65 years and older, and 23% was aged 45 to 64 years. Similar to Cascade County, the majority of residents were white (93%) or American Indian (3%). Both Cascade and Yellowstone counties are classified as rural counties — Cascade County has a population density of 29.8 persons per square mile, and Yellowstone County has a population density of 49.1 persons per square mile. These communities are served by three tertiary-care hospitals that provide comprehensive stroke diagnostic and treatment services. These facilities provide services for large multicounty areas that extend across state boundaries.

### Telephone survey

From February 2004 through April 2004, the Montana Department of Public Health and Human Services conducted a random-digit–dial telephone survey of adults aged 45 years and older living in Cascade (n = 400) and Yellowstone (n = 400) counties. Eligible persons living in households with more than one eligible respondent were randomly selected. A trained interview team using computer-assisted telephone interviewing software conducted the survey. The survey was field tested to detect potential problems with questions or answer categories and then revised as needed. A total of 3520 calls were made as part of the survey. Of these calls, 1002 (28%) were nonworking numbers, 754 (21%) were households with no eligible respondent, 426 (12%) were not private residences, and 252 (7%) were no answer/answering machine or busy. Of the remaining calls to persons in eligible households (n = 1086), 800 (74%) were completions, 224 (21%) were refusals, 39 (4%) were unable to complete due to communication/language barriers, and 23 (2%) were not completed because the eligible respondent was not available during the interviewing period. Up to 15 attempts were made to complete unanswered calls.

The survey included questions on the warning signs and risk factors for stroke, use of 911 EMS, previous diagnoses of risk factors for stroke, and demographic information. Open-ended questions adapted from Pancioli et al were used to assess respondents' knowledge of the warning signs and risk factors for stroke (2). Respondents were prompted to name up to three warning signs and three risk factors for stroke. Respondents were asked four questions adapted from Yoon and colleagues to identify what they would do if they witnessed someone having a stroke or if they experienced sudden stroke warning signs including numbness, paralysis, and speech problems that would not go away (10). Respondents were also asked a series of questions from the Behavioral Risk Factor Surveillance System Survey to identify if they had a history of heart attack, angina, coronary heart disease, stroke, transient ischemic attack (TIA), atrial fibrillation, diabetes, high blood pressure, or high cholesterol and if they currently smoked cigarettes (11). Respondents who reported a history of a heart attack, angina, or coronary heart disease were classified as having a history of heart disease. Female respondents who had been told only that they had gestational diabetes were not categorized as persons with a current diagnosis of diabetes. Respondents who reported that they smoked cigarettes every day or some days were categorized as current smokers.

Based on current recommendations from national organizations (12-15), we considered the following as established warning signs for stroke: dizziness, difficulty understanding speech or slurred speech, severe headache, problems with vision, weakness on one or both sides of body or face, numbness on one or both sides of body or face, trouble walking, loss of balance, or lack of coordination. We considered high blood pressure, high cholesterol, smoking, diabetes, heavy alcohol use, history of heart disease, and history of stroke or TIA to be established stroke risk factors.

### Data analyses

Data analyses were completed using SPSS V11.5 software (SPSS Inc, Chicago, Ill). Chi-square tests were used to compare differences in respondent knowledge of two or more warning signs, two or more risk factors for stroke, and use of 911 EMS by age, sex, and history of stroke risk factors. Multiple logistic regression analyses were conducted to identify demographic and self-reported risk factors independently associated with knowledge of warning signs and risk factors for stroke.

## Results

The mean age of respondents (N = 800) was 61 years (range 45 to 95); 60% were female; 96% were white; 2% were American Indian; and 93% reported 12 or more years of education. Ten percent reported a history of atrial fibrillation; 6% reported a history of diabetes; 37% reported a history of high blood pressure; 31% reported a history of high cholesterol; 17% currently smoked cigarettes; and 40% were former smokers. Eight percent reported a history of heart disease, and 6% reported a history of stroke or TIA. Overall, 80% reported one or more risk factors for stroke, and 56% reported two or more risk factors for stroke.

Numbness on any side of the face or body (45%) and speech problems (38%) were the most frequently reported established warning signs for stroke (Table 1). Fewer respondents reported vision problems (18%) or difficulty walking (11%). Overweight (56%), smoking (50%), and high blood pressure (44%) were the most frequently reported risk factors for stroke (Table 1).

The majority of respondents (70%) could identify two or more warning signs for stroke (Table 2). Women (75%) were more likely than men (62%) to identify two or more established warning signs for stroke, and respondents aged 45 to 64 years (76%) were more likely than those aged 65 years and older (59%) to identify two or more established warning signs for stroke. Just under half of the respondents (45%) could identify two or more established risk factors for stroke. Respondents aged 45 to 64 years (48%) were more likely to identify two or more established risk factors for stroke compared with those aged 65 years and older (40%).

Adjusting for multiple factors using logistic regression analyses, women, individuals aged 45 to 64 years, those with 12 or more years of education, and individuals with a history of high cholesterol were more likely to identify two or more established warning signs for stroke compared with respondents without these characteristics (Table 3). Women and respondents aged 45 to 64 years were more likely to identify two or more established risk factors for stroke compared with men and with respondents aged 65 years and older.

Overall, the majority of respondents (76%) indicated they would call 911 EMS if they witnessed someone having a stroke (Table 4). There were no differences by age, sex, or years of education in the proportion of respondents who indicated they would call 911 if they witnessed someone having a stroke (data not shown). When asked what they would do if they were experiencing sudden difficulty speaking, numbness, or weakness or paralysis, 43% to 49% of individuals indicated they would call 911. Depending on the symptom, 17% to 23% indicated they would go to the hospital, 14% to 19% would call their doctor, 11% to 18% would call their spouse or a family member, and 3% to 5% would do something else (Table 4). Respondents aged 65 years and older were more likely than respondents aged less than 65 to indicate they would call 911 if they experienced sudden difficulty speaking (49% vs 42%, *P* = .04), numbness (50% vs 40%, *P* = .006), or weakness or paralysis (55% vs 45%, *P* = .004). There were no differences by sex or years of education in the proportion of respondents who indicated they would call 911 if they experienced any of these warning signs (data not shown).

## Discussion

The majority of respondents from these rural counties were aware of the established warning signs for stroke, and awareness was higher in women, younger respondents, those with a higher level of education, and those with a history of high cholesterol compared with respondents without these characteristics. Interestingly, respondents with a history of other major stroke risk factors (e.g., high blood pressure) were no more aware of the warning signs compared with respondents without these conditions. Overall, fewer respondents were aware of the established risk factors for stroke. We also found that the majority of respondents would call 911 if they thought someone was having a stroke, but less than half would call 911 if they were experiencing stroke warning signs.

Individuals responding to the survey lived in communities that are typical of many communities across the United States, where health care for a large region is centered in a nearby town. Awareness of established stroke warning signs and risk factors was higher than awareness levels reported in other community surveys (10,16,17). In 1999, only 30% of adults surveyed in Michigan identified two or more warning signs correctly, and 34% identified two or more risk factors correctly (17). In 2000, more than 40% of respondents in Cincinnati, Ohio, identified two or more warning signs for stroke, and 32% identified two or more established risk factors for stroke (16). A study of adults living in an urban area of Australia in 1999 found that 26% of respondents to a telephone survey identified two or more warning signs for stroke, and 50% identified two or more risk factors for stroke (10).

Our findings on what respondents would do if they witnessed someone having a stroke or if they were experiencing warning signs of stroke are comparable to previous studies from Australia and Michigan (10,17). In Australia, 67% of respondents would call an ambulance if they witnessed someone having a stroke, while less than half of respondents would call an ambulance if they were experiencing sudden stroke warning signs (45% difficulties with speech, 38% numbness/weakness, 35% weakness or paralysis). In Michigan, however, 79% indicated they would call 911 if someone was having a stroke.

There are a number of limitations to this study. First, the survey does not reflect the experience of residents without telephones. Second, self-reported information regarding risk factors for stroke is subject to recall bias. Previous studies, however, have found that self-reported risk factors for cardiovascular disease are reported reliably (18,19). Third, respondents were asked unaided questions to assess respondent knowledge of the warning signs and risk factors for stroke. Some previous studies assessing awareness of stroke warning signs used aided questions and found higher levels of knowledge than the levels found in this study (20). It is possible that unaided questions may underestimate the awareness of the warning signs of stroke, and aided questions may overestimate awareness. Fourth, this study was conducted in a rural non-Hispanic white population, and there may be significant variation in awareness of stroke warning signs and risk factors in other geographic and racial and ethnic communities in the United States.   

Our findings about stroke awareness in rural communities are important because they are similar to studies published from academic centers working in urban areas (10,16,17). This effort, however, represents a collaboration between a state public health agency and several regional health care systems not only to understand the levels of stroke awareness in two communities at baseline but also to promote community awareness and increased use of EMS and to define regional approaches for prompt stroke treatment in cooperation with the stroke referral centers in these counties. Based on the findings reported here, the Montana Cardiovascular Health Program and these partners have developed and implemented a multifaceted intervention to increase community awareness of the warning signs of stroke and the need to use 911 adapting strategies that have been shown to be successful (8,21).

It is reassuring that levels of community awareness and knowledge about stroke in rural settings are not markedly different from levels found in urban environments. Others have shown that multifaceted interventions to increase community awareness, use of EMS, and availability of prompt diagnosis and treatment can succeed (7,8). Strategies to reduce barriers to prompt stroke treatment may be somewhat different across large frontier areas compared with urban environments. Cooperative efforts between public health agencies, communities, and prehospital and acute care systems, however, can build on the published experience of others to understand and improve knowledge about stroke warning signs and risk factors as part of broader public health interventions targeted to stroke prevention and treatment. In the United States, public health programs are only beginning to develop partnerships and work toward developing and documenting successful interventions in a wide variety of settings. This report represents the beginning of efforts to meet the challenges in rural communities in Montana and across many rural areas.

## Figures and Tables

**Table 1:** Perceptions of Stroke Warning Signs and Risk Factors Among Survey Respondents Aged ⩾45 Years (N = 800), Montana, 2004^a^

**Responses**	**No. (%)**

**Warning signs**	

Numbness (any side of face or body)^b^	360 (45)
Speech difficulties^b^	300 (38)
Do not know	306 (38)
Dizziness^b^	278 (35)
Headache^b^	204 (26)
Weakness (any)^b^	198 (25)
Other	174 (22)
Vision problems^b^	140 (18)
Shortness of breath	108 (14)
Difficulties walking^b^	85 (11)

**Risk factors**	

Overweight	445 (56)
Smoking^b^	400 (50)
High blood pressure^b^	351 (44)
Lack of exercise	208 (26)
High cholesterol^b^	172 (22)
Do not know	169 (21)
Stress	155 (19)
Alcohol use^b^	101 (13)
Other	96 (12)
Family history of heart disease	58 (7)
Diabetes^b^	57 (7)
Family history of stroke	45 (6)
History of heart disease^b^	40 (5)

a Only warning signs and risk factors with at least 5% responding are listed.

b Established warning signs and risk factors.

**Table 2:** Knowledge of Established Stroke Warning Signs and Risk Factors, Overall and by Age and Sex, Montana, 2004

	**Total** **(N = 800)** **No. (%)**	**Male** **(n = 322)** **No. (%)**	**Female** **(n = 478)** **No. (%)**	**Aged 45–64** **years** **(n = 511)** **No. (%)**	**Aged 65+ ** **years** **(n = 287)** **No. (%)**

**Number of warning signs**					

One or more	697 (87)	272 (85)	425 (89)	461 (90)^a^	234 (82)
Two or more	557 (70)	200 (62)	357 (75)^a^	387 (76)^a^	169 (59)
Three	311 (39)	111 (35)	200 (42)^a^	225 (44)^a^	85 (30)

**Number of risk factors**					

One or more	683 (85)	262 (81)	421 (88)^a^	443 (87)	238 (83)
Two or more	360 (45)	133 (41)	227 (48)	243 (48)^a^	116 (40)
Three	55 (7)	16 (5)	39 (8)	41 (8)	14 (5)

a P ⩽.05 for comparisons by sex and age category.

**Table 3:** Factors Independently Associated With Awareness of Two or More Warning Signs and Risk Factors for Stroke Among Respondents Aged ⩾45 Years^a^

**Factor**	**Odds Ratio (95% Confidence Interval)**

**Knowledge of two or more warning signs**	

Sex (female)	2.02 (1.46-2.80)
Age (45-64 years)	2.44 (1.78-3.46)
Education level (⩾12 years)	1.96 (1.08-3.56)
Atrial fibrillation	0.77 (0.46-1.29)
Diabetes	0.92 (0.48-1.76)
High blood pressure	0.94 (0.67-1.33)
High cholesterol	1.68 (1.17-2.42)
History of heart disease^b^	1.54 (0.84-2.83)
History of stroke or transient ischemic attack	1.20 (0.62-2.32)
Current smoker	1.00 (0.63-1.59)
Former smoker	1.13 (0.79-1.61)

**Knowledge of two or more risk factors**	

Sex (female)	1.48 (1.07-2.05)
Age (45-64 years)	1.35 (1.01-1.81)
Education level (⩾12 years)	0.75 (0.41-1.35)
Atrial fibrillation	0.75 (0.46-1.22)
Diabetes	1.50 (0.82-2.74)
High blood pressure	1.19 (0.87-1.62)
High cholesterol	1.11 (0.81-1.52)
History of heart disease^b^	1.23 (0.71-2.10)
History of stroke or transient ischemic attack	1.39 (0.76-2.52)
Current smoker	1.18 (0.78-1.78)
Former smoker	1.16 (0.85-1.60)

a Referent groups include the following: males, respondents aged ⩾65 years, respondents with less than 12 years of education, respondents without a history of atrial fibrillation, diabetes, high blood pressure, high cholesterol, heart disease, stroke, or transient ischemic attack, and respondents who reported never smoking cigarettes.

b Includes heart attack, angina, or coronary heart disease.

**Table 4:** Reactions to Witnessing a Potential Stroke and to Experiencing Potential Warning Signs of a Stroke Among Survey Respondents Aged ⩾45 years, Montana, 2004

	**If you thought someone was having a stroke, what is the first thing you would do?** **No. (%)**	**If you experienced sudden . . . that would not go away, what is the first thing you would do?**		

		**Difficulty speaking** **No. (%)**	**Numbness, tingling, or dead sensation** **No. (%)**	**Weakness or paralysis** **No. (%)**
**Take them/go to hospital**	121 (15)	132 (17)	176 (22)	186 (23)
**Tell them/call your/their doctor**	21 (3)	121 (15)	150 (19)	110 (14)
**Call 911**	608 (76)	354 (44)	346 (43)	388 (49)
**Tell them/call spouse or family member**	4 (1)	147 (18)	92 (12)	88 (11)
**Do something else**	38 (5)	38 (5)	32 (4)	25 (3)
**Do not know**	8 (1)	8 (1)	4 (1)	3 (0)
